# Redescription of the rare, incompletely described *Mongolodiaptomus rarus* (Ranga Reddy, Sanoamuang & Dumont, 1998) (Copepoda, Calanoida, Diaptomidae), from freshwater habitats in northeast Thailand, with comments on conservation status

**DOI:** 10.7717/peerj.21683

**Published:** 2026-09-11

**Authors:** Laorsri Sanoamuang, Prapatsorn Dabseepai, Kamonwan Koompoot

**Affiliations:** 1 Laboratory of Biodiversity and Environmental Management, International College, Khon Kaen University, Khon Kaen, Thailand; 2 Applied Taxonomic Research Center, Faculty of Science, Khon Kaen University, Khon Kaen, Thailand

**Keywords:** Biodiversity, Freshwater Biology, Taxonomy, Zoology, Copepods, Crustacea, Diaptomidae, Sustainability, Endemic, Temporary-water Habitats

## Abstract

The genus *Mongolodiaptomus* Kiefer currently contains 17 species, distributed across Asia, predominantly occurring in countries in the lower Mekong River Basin. Herein, *Mongolodiaptomus rarus* (Ranga Reddy, Sanoamuang & Dumont, 1998), an incompletely described species, is redescribed based on specimens from Thailand. Morphologically, it belongs to the *M. gladiolus* species group, closely similar to *M. calcarus* (Shen & Tai, 1965), *M. dumonti* Sanoamuang, 2001, and *M. gladiolus* (Shen & Lee, 1963). *M*. *rarus* is, however, distinctly different from its congeners, in particular the prolonged oval shape of the male right leg 5-second exopod and the position and shape of the principal lateral spine on this segment. *Mongolodiaptomus rarus* occurs mostly in temporary-water habitats, although it also inhabits swamps, which are permanent-water habitats. It appears to be rare, restricted to a few localities, and has to date been recorded from 20 sites in five provinces. Currently, *M. rarus* is endemic to northeast Thailand. It was found during May and September, over a temperature range of 25–38 °C, pH 6.6–8.5, and conductivity of 2–299 µS cm^−1^. The recent development of modern highway construction and the introduction of many agricultural crops have resulted in the disappearance of their habitats, so conservation of this rare taxon is proposed.

## Introduction

Copepods, one of the most diversified groups of microcrustaceans, commonly occur in freshwater and marine environments (Marrone et al., 2017). Free-living copepods are classified into three groups based on their morphological features: Calanoida, Cyclopoida, and Harpacticoida. Members of the Diaptomidae family in the Calanoida order have significant success in various freshwater environments, with more than 440 species (Boxshall & Defaye, 2008). Ninety-two species within the 22 genera of diaptomid copepods have been recorded in the Oriental region, which includes Southeast Asia, South Asia, and parts of East Asia; among these, 81 species within the 10 genera are endemic to this region (Boxshall & Defaye, 2008). In Thailand, the diaptomid genera *Mongolodiaptomus* Kiefer, 1937, and *Tropodiaptomus* Kiefer, 1932, are the most diverse, each comprising eleven recognized species (Sanoamuang & Koompoot, 2024; Koompoot & Sanoamuang, 2025).

The freshwater diaptomid copepod genus *Mongolodiaptomus* is extensively distributed across Asia; however, it is absent from India (Kulkarni & Pai, 2016). To date, 17 valid species have been recorded worldwide (Chaicharoen & Sanoamuang, 2025), predominantly located in the lower Mekong River Basin, including the mainland Southeast Asian countries such as Thailand, Vietnam, Cambodia, and Laos (Watiroyram & Sanoamuang, 2017; Sanoamuang & Watiroyram, 2018; Sanoamuang & Dabseepai, 2021; Boonmak & Sanoamuang, 2022; Chaicharoen & Sanoamuang, 2022; Sanoamuang & Koompoot, 2024). Certain documented species are also found in Malaysia, Singapore, the Philippines, Indonesia, China, and Taiwan (Alekseev et al., 2013; Lim & Lai, 2014; Alekseev, Yusoff & Fefilova, 2016; Lopez et al., 2017; Li et al., 2018; Lee et al., 2021).

Kiefer (1932) originally classified the genus *Mongolodiaptomus* as a subgenus of *Eudiaptomus* and subsequently upgraded it to generic status, with *Mongolodiaptomus formosanus* Kiefer, 1937, as the type species. Ranga Reddy, Sanoamuang & Dumont (2000) considered the ornamentation on the right second exopod of the male fifth legs as an important characteristic for differentiating diaptomid copepods, especially within the closely related genera *Neodiaptomus* Kiefer, 1932; *Allodiaptomus* Kiefer, 1936; and *Mongolodiaptomus*. The classification system used to distinguish the genus *Mongolodiaptomus* from similar genera is detailed in Sanoamuang & Koompoot (2024).

In Thailand and Vietnam, 11 and nine species have to date been recorded, respectively, making this region the most diverse hotspot for *Mongolodiaptomus* taxa compared to six species each in China and Cambodia, as well as four species in Laos (Tran et al., 2016; Li et al., 2018; Boonmak & Sanoamuang, 2022; Chaicharoen & Sanoamuang, 2022; Chaicharoen & Sanoamuang, 2025; Wei et al., 2023; Sanoamuang & Koompoot, 2024). Among these, *Mongolodiaptomus rarus* (Ranga Reddy, Sanoamuang & Dumont, 1998) was originally described as *Allodiaptomus rarus* by Ranga Reddy, Sanoamuang & Dumont (1998), based on a single male specimen collected from an unspecified freshwater body in Thailand. This species has been transferred to the genus *Mongolodiaptomus* by Sanoamuang (2001), Sanoamuang & Athibai (2002), and Sanoamuang & Watiroyram (2019). In this study, we present a full morphological redescription of *M. rarus*, collected from 20 freshwater habitats in Thailand, based on adult male and female specimens.

The electronic version of this article in Portable Document Format (PDF) will represent a published work according to the International Commission on Zoological Nomenclature (ICZN), and hence the new names contained in the electronic version are effectively published under that Code from the electronic edition alone. This published work and the nomenclatural acts it contains have been registered in ZooBank, the online registration system for the ICZN. The ZooBank LSIDs (Life Science Identifiers) can be resolved and the associated information viewed through any standard web browser by appending the LSID to the prefix “http://zoobank.org”. The LSID for this publication is: urn:lsid:zoobank.org:pub:9BB5F803-0946-4893-94B5-9CF437C17AAA. The online version of this work is archived and available from the following digital repositories: PeerJ, PubMed Central SCIE and CLOCKSS.

## Materials and Methods

**Figure 1:**
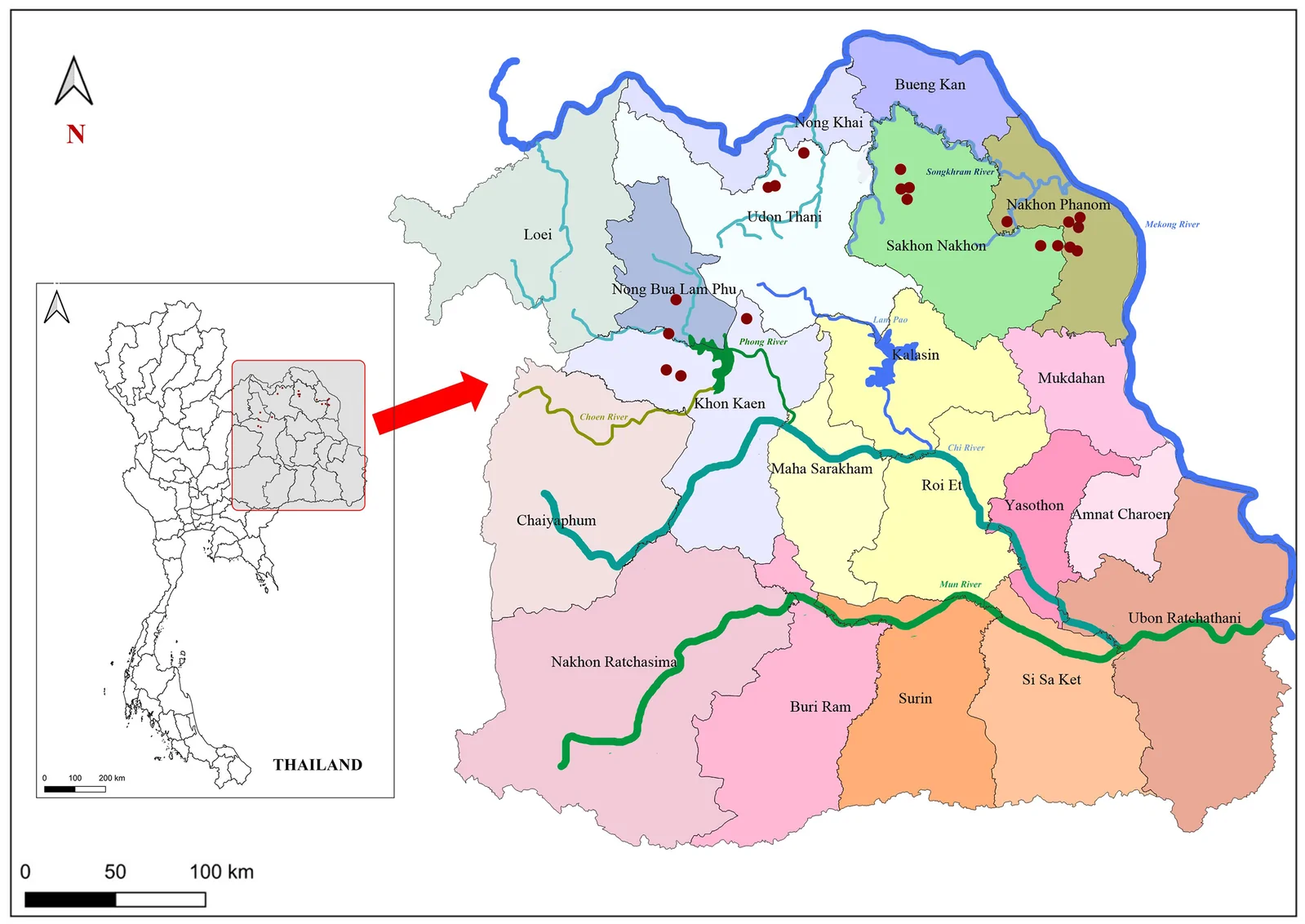
Map of northeast Thailand with major rivers and 20 provinces. The red spots are sampling sites of *Mongolodiaptomus rarus*. This map was created by the program QGIS.

Samples were collected qualitatively from a wide variety of freshwater habitats in northeast Thailand during June 1993 and August 2007 (Table 1 and Fig. 1), using a plankton net with a mesh size of 60 µm. The concentrated samples were then preserved in 70% ethanol or 4% formaldehyde immediately after collection. Specimens were cut and affixed at 40–100× magnification using an Olympus SZ40 stereomicroscope. For drawings, the habitus and all appendages were dissected and rendered at 400× and 1,000× magnification using a camera lucida drawing tube affixed to an Olympus CH30 compound microscope. The CorelDRAW Graphics Suite 2017 program was employed for the final version of the illustrated figures.

Due to only one dissected male of *Mongolodiaptomus rarus*, collected from an unspecified freshwater body in northeast Thailand, mounted in glycerol on a single slide, designated the holotype, and deposited in the British Museum (Natural History), London, under the registration number 1995.941 (Ranga Reddy, Sanoamuang & Dumont, 1998). No paratypes and no description of female morphological features are available. We, therefore, redescribed male and female morphological characters of this species, based on specimens collected from northeast Thailand, which is the same area as the holotype specimen.

Specimens for scanning electron microscopy (SEM) were prepared as previously described in Sanoamuang & Dabseepai (2021). The subsequent abbreviations are employed in both the content and the figures: **ae**, aesthetasc; **Enp**, endopod; **Enp-n**, endopodal segment n; **Exp**, exopod; **Exp-n**, exopodal segment n**; Pdg1–Pdg5**, pedigers 1–5; **P1–P5**, legs 1–5; **sp**, spine. The nomenclature and descriptive terms adhere to the standards set by Huys & Boxshall (1991).

## Results

### Taxonomy

**Table:** 

Infraclass Neocopepoda Huys & Boxshall, 1991
Order Calanoida Sars, 1903
Family Diaptomidae Baird, 1850
Sub-family Diaptominae Kiefer, 1932
**Genus** ***Mongolodiaptomus*** **Kiefer, 1937**

**Type species**. *Mongolodiaptomus formosanus* Kiefer, 1937, by original designation.

**Table:** 

***Mongolodiaptomus rarus*(Ranga Reddy, Sanoamuang & Dumont, 1998)**Figures 2–11

*Allodiaptomus rarus* Ranga Reddy, Sanoamuang & Dumont, 1998: 220–222, figs. 95–102; in Sanoamuang (1999): 218–219.

**Table 1:** Sampling sites of *Mongolodiaptomus rarus* in northeast Thailand, with information on type of habitats, sampling dates, locations, water variables, and diaptomid species co-occurrence. The following abbreviations are as follows: Temp. = temperature, Cond. = conductivity.

No.	Type of habitat	Sampling date	Location			Water variables			Co-occurrence with other diaptomid copepods	Reference
			District	Province	Coordinates	Temp. (^∘^)	pH	Cond. (µS cm^−1^)		
1	Swamp	22/08/2007	Wiang Kao	Khon Kaen	16°38′43.77″N, 102°18′11.90″E	31.2	8.2	299	*Mongolodiaptomus phutakaensis, Phyllodiaptomus praedictus*	Sanoamuang & Koompoot (2024)
2	Temporary pond	06/06/1993	Muang	Nong Bua Lam Phu	17°03′53.89″N, 102°21′49.18″E	34	8.0	200	*Mongolodiaptomus dumonti, P. praedictus*	Sanoamuang (2001)
3	Roadside canal	07/05/1999	Kusuman	Sakon Nakhon	17°19′01.30″N, 104°17′19.20″E	28.5	7.7	65	*Neodiaptomus laii, N. songkramensis, Tropodiaptomus oryzanus, Vietodiaptomus blachei*	Sanoamuang & Athibai (2002)
4	Rice paddy	20/05/2003	Charoen Sin	Sakon Nakhon	17°43′34.07″N, 103°32′31.59″E	–	7.4	–	No other species present	This study
5	Rice paddy	20/05/2003	Kusuman	Sakon Nakhon	17°19′22.71″N, 104°22′57.52″E	–	7.0	–	*Eodiaptomus phuphanensis, Mongolodiaptomus calcarus, Neodiaptomus yangtsekiangensis*	This study
6	Temporary pond	15/05/1999	Muang	Nakhon Phanom	17°17′32.09″N, 104°28′17.97″E	29	7.5	90	*Vietodiaptomus blachei*	This study
7	Temporary pond	15/05/1999	Muang	Nakhon Phanom	17°18′09.60″N, 104°26′47.44″E	29	7.1	35	No other species present	This study
8	Roadside canal	24/05/1999	Phon Sawan	Nakhon Phanom	17°26′44.98″N, 104°26′27.66″E	30	7.5	85	No other species present	This study
9	Temporary pond	17/05/2000	Na Wa	Nakhon Phanom	17°27′04.18″N, 104°06′17.82″E	25	8.5	55	*Heliodiaptomus phuthaiorum, Neodiaptomus yangtsekiangensis, Phyllodiaptomus praedictus*	This study
10	Small pool	18/05/2000	Phon Sawan	Nakhon Phanom	17°26′52.67″N, 104°26′41.19″E	28	7.3	65	*Vietodiaptomus blachei*	This study
11	Temporary pond	18/05/2000	Phon Sawan	Nakhon Phanom	17°28′42.79″N, 104°29′42.06″E	29	6.9	65	*Vietodiaptomus blachei, Neodiaptomus songkhramensis*	This study
12	Small pool	01/09/2003	Charoen Sin	Sakon Nakhon	17°33′36.09″N, 103°34′36.63″E	35	7.5	48	*Phyllodiaptomus praedictus*	This study
13	Reservoir	01/09/2003	Charoen Sin	Sakon Nakhon	17°37′16.88″N, 103°33′09.61″E	32	7.3	14	*Mongolodiaptomus botulifer*	This study
14	Reservoir	01/09/2003	Charoen Sin	Sakon Nakhon	17°37′23.38″N, 103°35′32.25″E	33	7.9	2	No other species present	This study
15	Rice paddy	29/04/2000	Nong Na Kham	Khon Kaen	16°50′26.85″N, 102°18′40.41″E	38	6.6	75	*Dentodiaptomus javanus, Eodiaptomus sanoamuangae, Mongolodiaptomus calcarus, Phyllodiaptomus praedictus*	This study
16	Roadside canal	29/04/2000	Phu Wiang	Khon Kaen	16°36′51.02″N, 102°22′44.26″E	35	7.3	100	*Phyllodiaptomus praedictus, Vietodiaptomus blachei*	This study
17	Roadside canal	20/05/2000	Ubonrat	Khon Kaen	16°55′43.15″N, 102°43′43.22″E	30	7.6	150	*Phyllodiaptomus praedictus, Vietodiaptomus blachei*	This study
18	Rice paddy	06/05/2000	Phen	Udon Thani	17°37′31.85″N, 102°53′17.76″E	26	7.5	110	*Dentodiaptomus javanus, Mongolodiaptomus calcarus, Neodiaptomus songkramensis, Phyllodiaptomus praedictus*	Sanoamuang & Athibai (2002)
19	Roadside canal	06/05/2000	Sang Khom	Udon Thani	17°48′36.76″N, 103°02′36.93″E	26	7.1	200	*Mongolodiaptomus botulifer, M. calcarus, Neodiaptomus songkramensis, Phyllodiaptomus praedictus, Vietodiaptomus blachei*	Sanoamuang & Athibai (2002)
20	Temporary pond	21/07/2004	Phen	Udon Thani	17°39′27″N, 102°52′52″E	28.1	6.7	0.015	No other species present	This study

**Figure 2:**
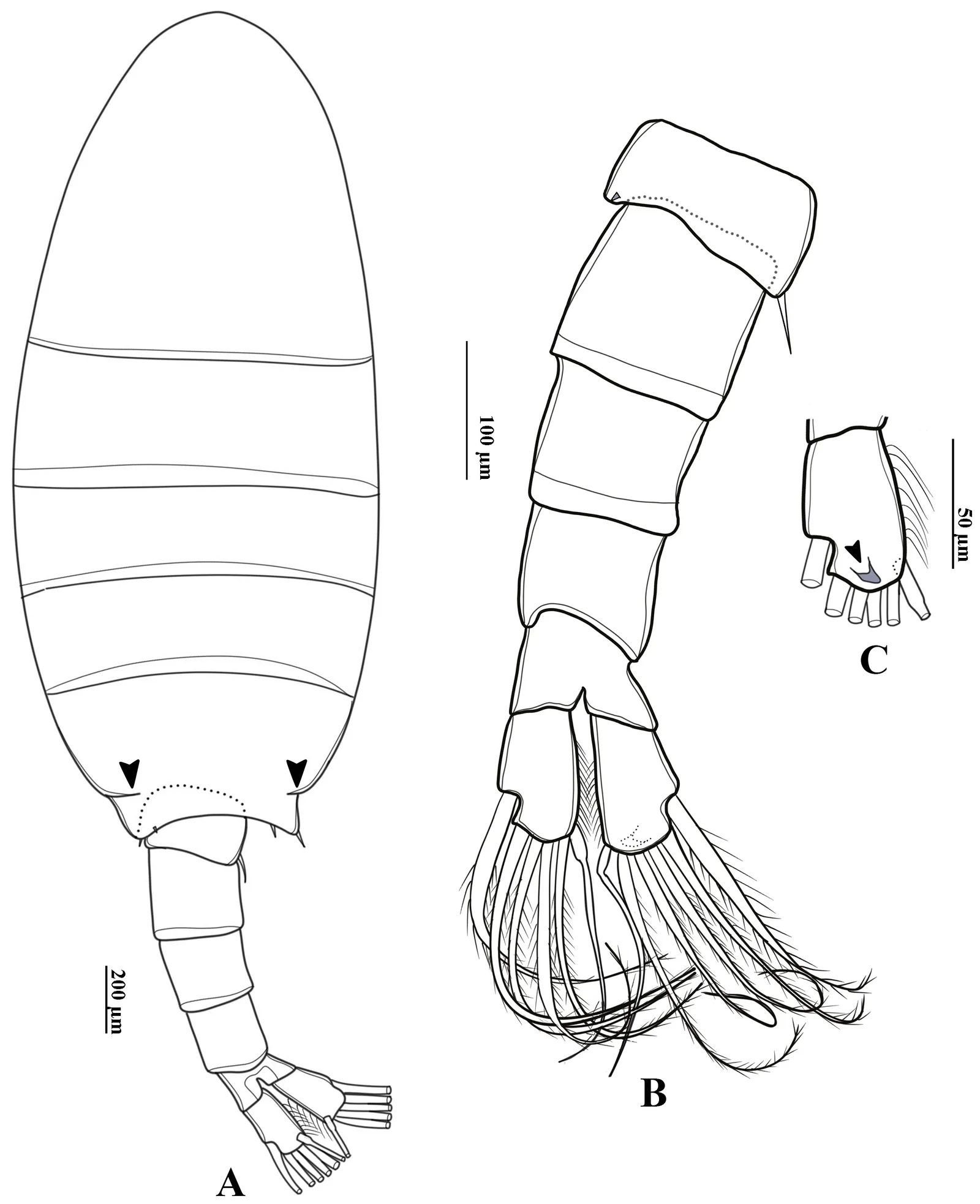
*Mongolodiaptomus rarus*, male. (A) Habitus, dorsal (arrows point to septa at lateral margins between Pdg4 and Pdg5); (B) urosome, dorsal; (C) right caudal ramus, ventral (arrow points to a small triangular-shaped chitinous tooth).

**Figure 3:**
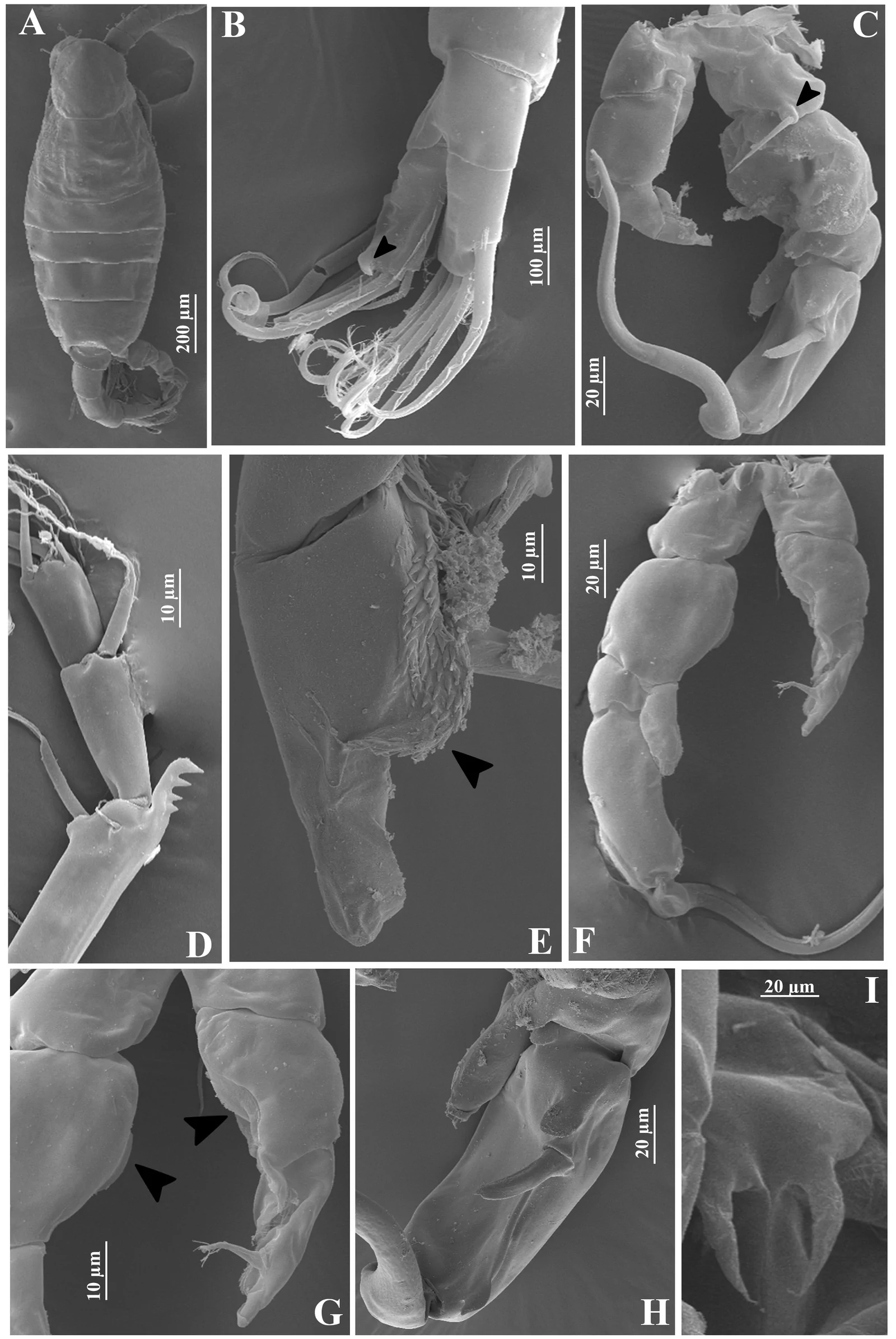
*Mongolodiaptomus rarus*, SEM photographs of males. (A) Habitus, dorsal; (B) last urosomite and caudal rami, lateral (arrow points to a small triangular-shaped chitinous tooth); (C) P5, posterior (arrow points to a coxal spine); (D) right antennule, segments 20–22; (E) distal part of left P5, posterior (black arrow points to the specialized crescent-shaped lobe of spinules); (F) P5, anterior; (G) distal part of left P5 and middle part of right P5, anterior (black arrows point to the hyaline membranes); (H) right P5 Exp-1, Exp-2, and Enp, posterior; (I) rostrum.

*Mongolodiaptomus rarus* (Ranga Reddy, Sanoamuang & Dumont, 1998) in Sanoamuang (2001): 41, 51, tab. 1; Sanoamuang (2002): 138–141, figs. 6-47, 6-48; Sanoamuang & Athibai (2002): 81, tab. 1; Sanoamuang (2004): 396; Watiroyram & Sanoamuang (2017): 16, 28–30; Sanoamuang & Watiroyram (2018): 793–794, tab. 2; Sanoamuang & Watiroyram (2019): 185–199; Sanoamuang & Dabseepai (2021): 5, 7, 16, 20, tab. 2 & 4; Sanoamuang & Koompoot (2024): 20, 31–39, tabs. 5–6.

**Figure 4:**
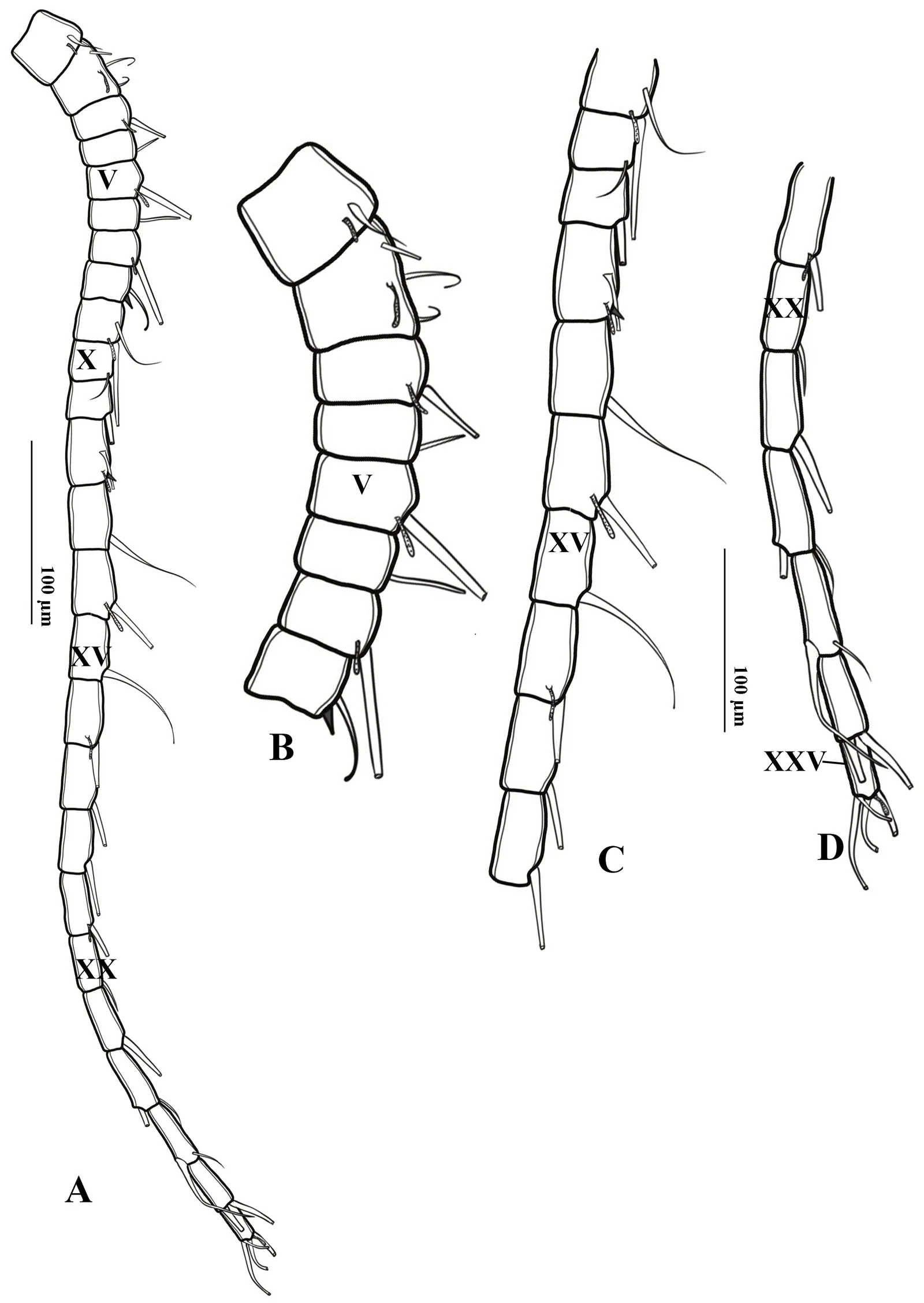
*Mongolodiaptomus rarus*, male left antennule. (A) Segments 1–25; (B) segments 1–8; (C) segments 9–18; (D) segments 19–25.

**Figure 5:**
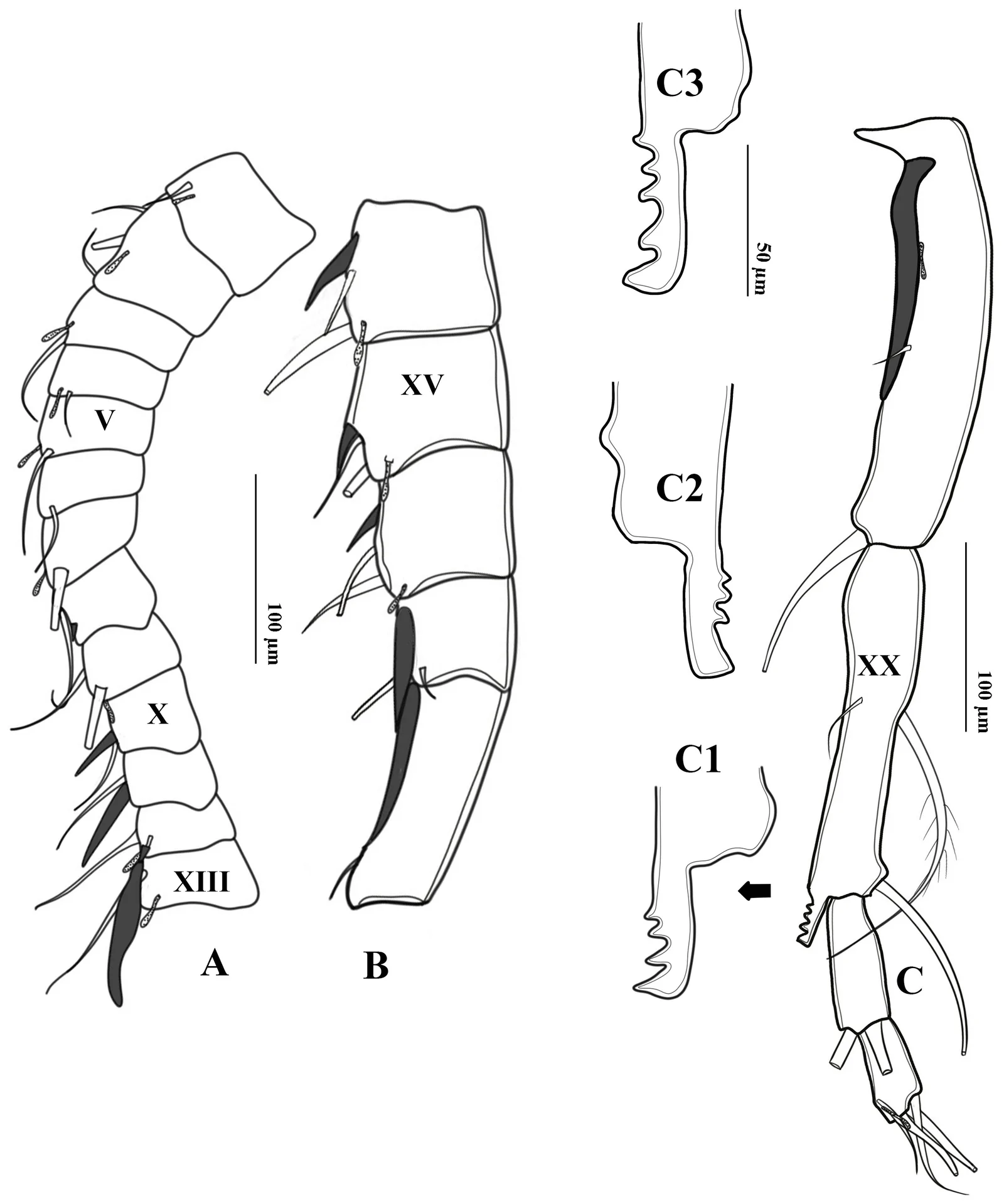
*Mongolodiaptomus rarus*, male right antennule. (A) Segments 1–13; (B) segments 14–18; (C) segments 19–22; (C1–C3), combs on antepenultimate segment.

### Localities and material examined.

Twenty sampling sites of *M. rarus* in northeast Thailand, with information on type of habitats, sampling dates, locations, water variables, and Diaptomid species co-occurrence, are presented in Table 1 and Fig. 1. In addition, 11 males and 11 females of *M. rarus* are deposited at the Thailand Natural History Museum (THNHM), accession numbers THNHM-lv-20177–20179. All specimens were collected from a swamp at the Kok Phutaka community forest, Muang Kao Phatthana Subdistrict, Wiang Kao District, Khon Kaen Province, northeast Thailand (16°38′43.77″N, 102°18′11.90″E); elevation 220 m a.s.l.; water temperature 31.2 °C; pH 8.2; conductivity 299 μS cm^−^^1^; on 22 April 2007 by P. Dabseepai and K. Koompoot.

**Figure 6:**
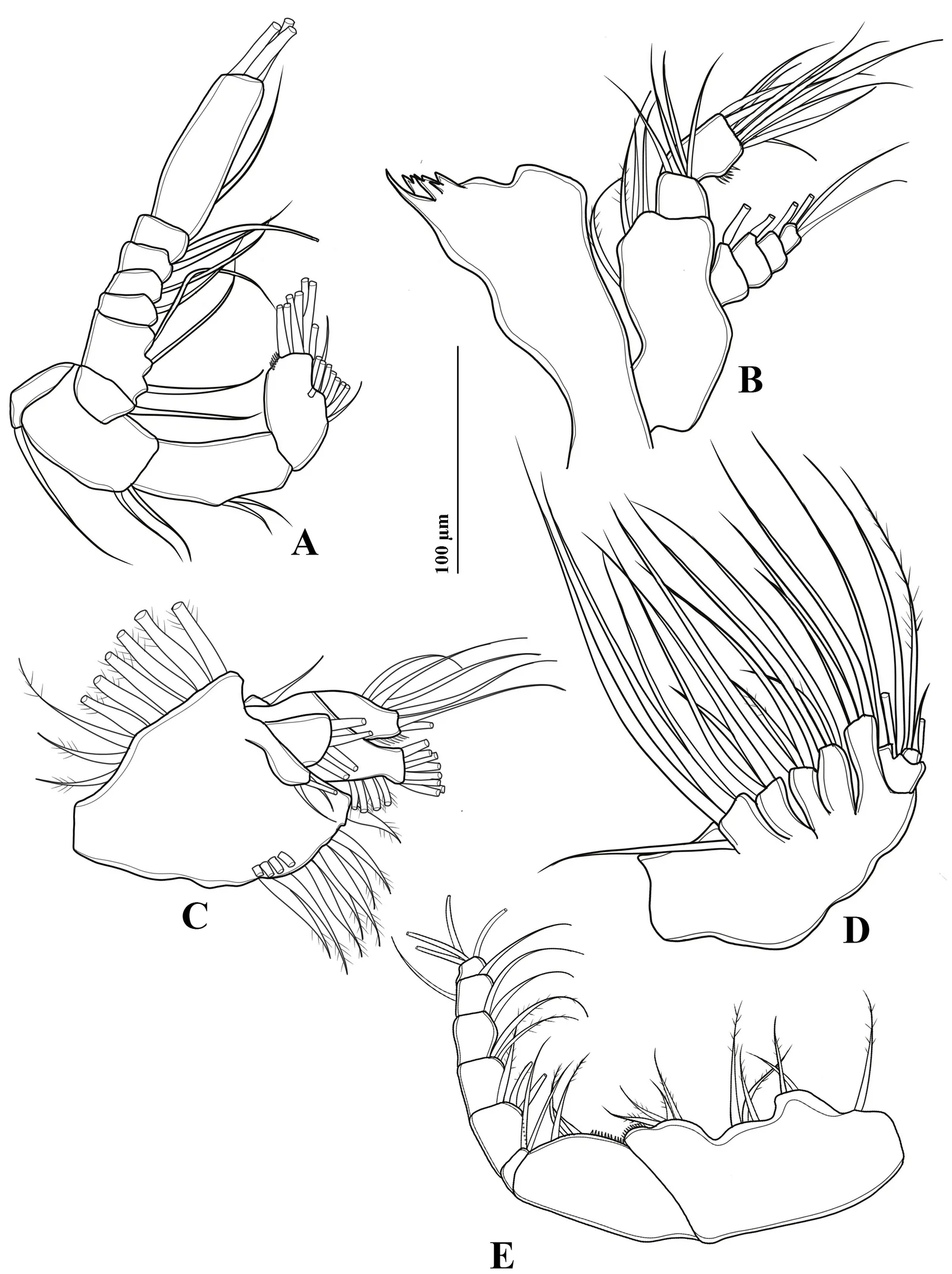
*Mongolodiaptomus rarus*, male mouthparts. (A) Antenna; (B) mandible; (C) maxillule; (D) maxilla; (E) maxilliped.

**Figure 7:**
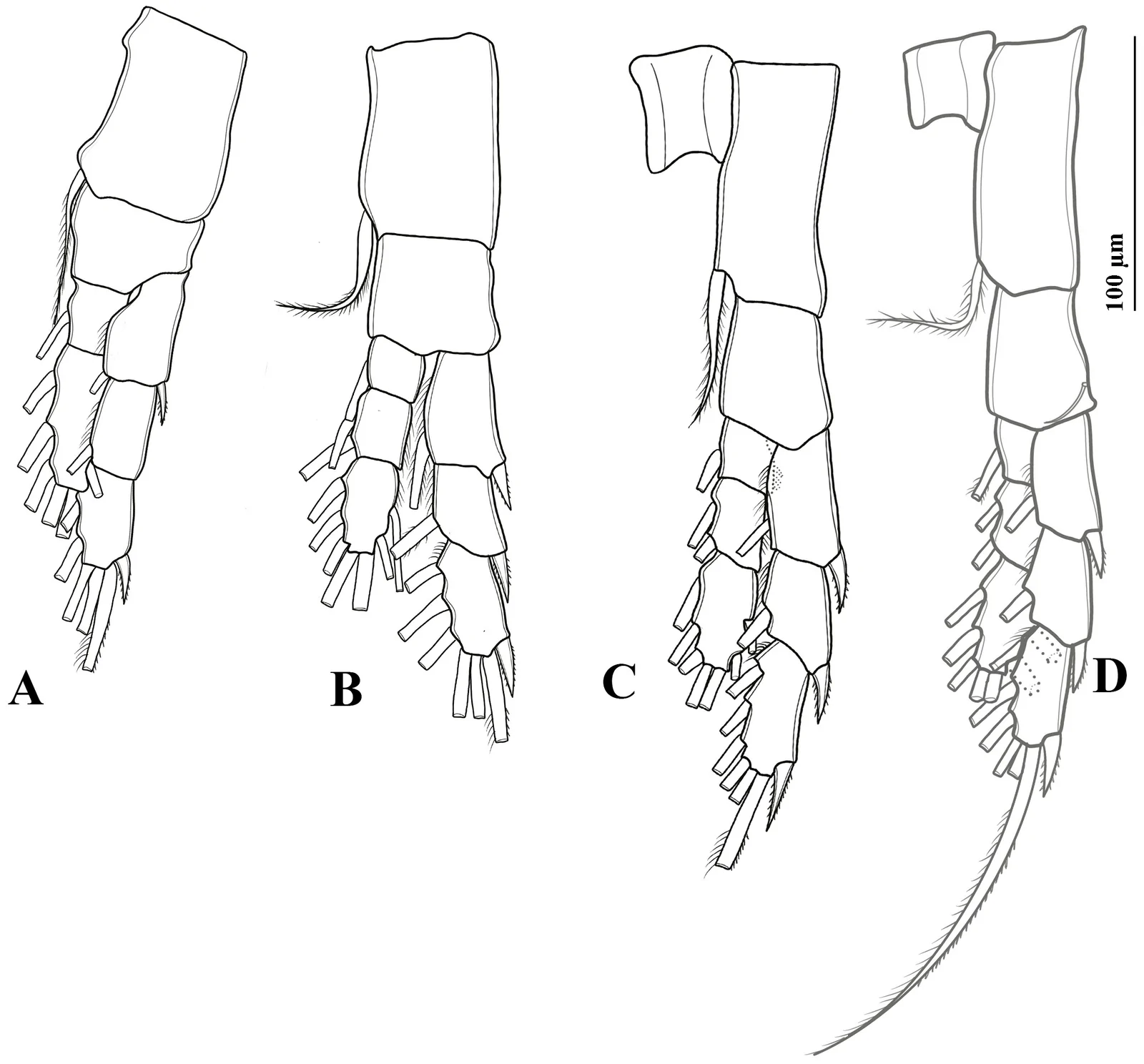
*Mongolodiaptomus rarus*, male swimming legs. (A) P1; (B) P2; (C) P3; (D) P4.

**Figure 8:**
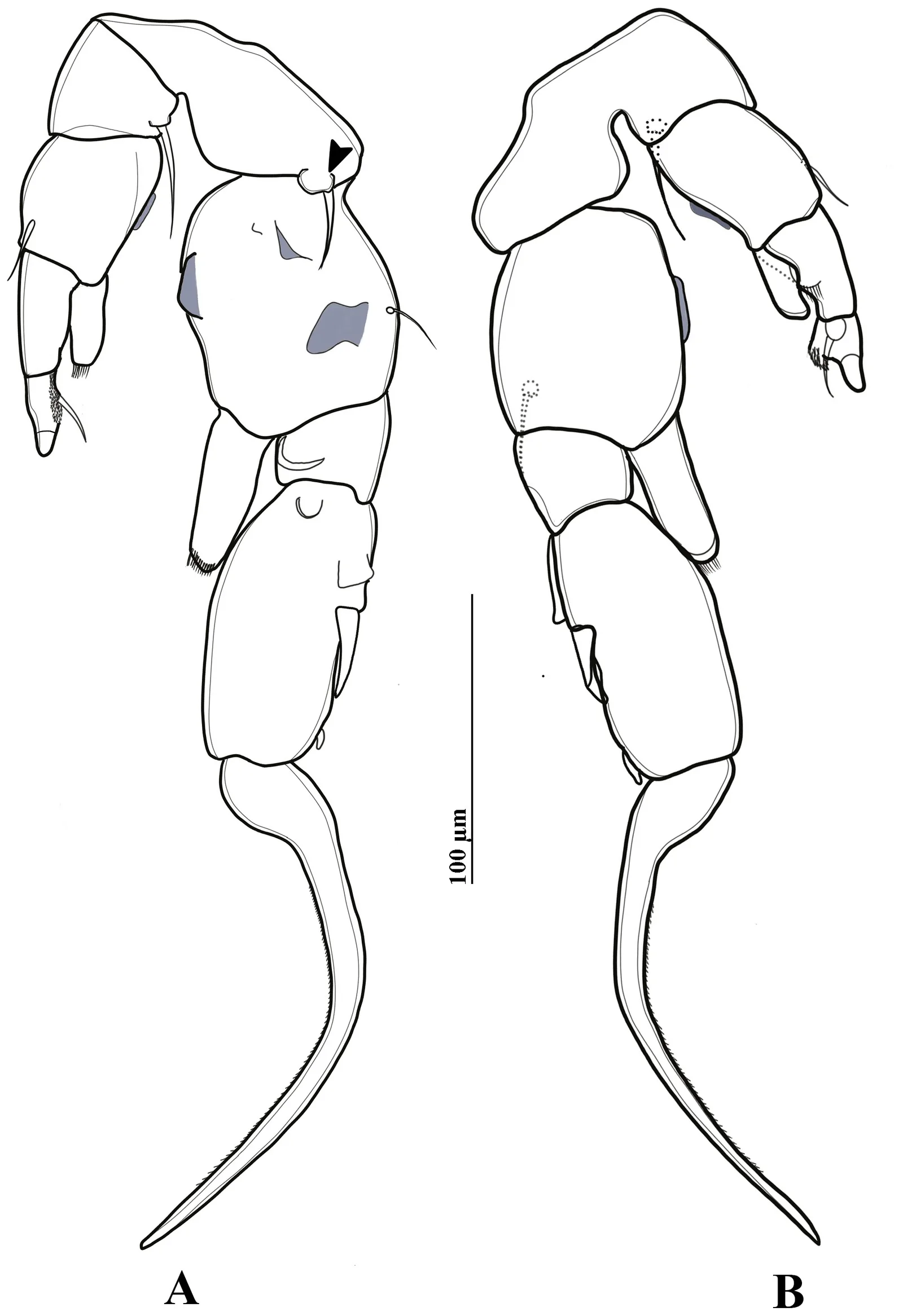
*Mongolodiaptomus rarus*, male. (A) P5, posterior (arrow points to a coxal spine); (B) P5, anterior.

**Description of adult male.** Total body length, measured from anterior margin of rostrum to posterior margin of caudal rami, 1.26–1.31 mm (mean = 1.29 mm, *n* = 5) (Figs. 1–8). Body smaller and slender than in female. Prosome about 2.3 times as long as urosome (Fig. 2A). Rostrum well-developed, with two spiniform processes (Fig. 3I). Fourth and fifth pedigerous somites partially fused except at lateral margins (Fig. 2A, arrows). Lateral wings of Pdg5 small and asymmetrical; right postero-lateral wing triangular, and left wing rounded; both wings with one tiny spine at distal corner and one inner sensillum-like spine; spines on right wing larger than spines on left wing (Fig. 2A).

Urosome (Figs. 2A, 2B, 3A) with five somites, slightly bent to right side. Genital somite asymmetrical, wider than other urosomites, right distal corner with one slender spine, about 0.5 times as long as wide (Fig. 2B). Urosomites 2–4 about as long as wide. Urosomites 2 and 3 without hairs on ventral side. Urosomite 4 asymmetrical, with expanded right dorso-posterior corner. Anal somite asymmetrical, left side slightly larger than right side. Caudal rami asymmetrical, right ramus slightly longer than left one, each ramus about 1.7 times as long as wide, inner margins hairy (Fig. 2B). Ventral surface of right caudal ramus armed with a small triangular-shaped chitinous tooth, located at distal middle surface, near bases of principal caudal setae (Figs. 2C, 3B, arrow). Each ramus armed with five plumose caudal setae and one base dorsal seta.

Antennules: asymmetrical, long, reaching to posterior end of genital somite. Left antennule (Figs. 4A–4D): 25-segmented. Armature formulae as in Table 2. Right antennule geniculated (Figs. 5A–5C), consisting of 22 segments, strongly dilated between segment XIII and segment XVIII. Segment XIII with largest strong spinous process, one seta, and one aesthetasc. Antepenultimate segment (segment XX) much longer than next segment. Spinous process on antepenultimate segment short, comb-like, with four or five teeth (Figs. 3D, 5C1–5C3), reaching 1/3 of next segment (Figs. 3D, 5C). Armature formulae as in Table 3.

Antenna (Fig. 6A) biramous. Coxa and basis with one and two bare setae on inner distal corner, respectively. Enp two-segmented; Enp-1 with two median setae along inner margin; Enp-2 with nine setae along inner margin, and seven setae apically; all setae bare. Exp seven-segmented, longer than Enp. Exp-1–6 with setal formula: 1, 3, 1, 1, 1, 1 along inner margin. Exp-7 with one seta on inner margin and three setae apically; all setae bare.

**Figure 9:**
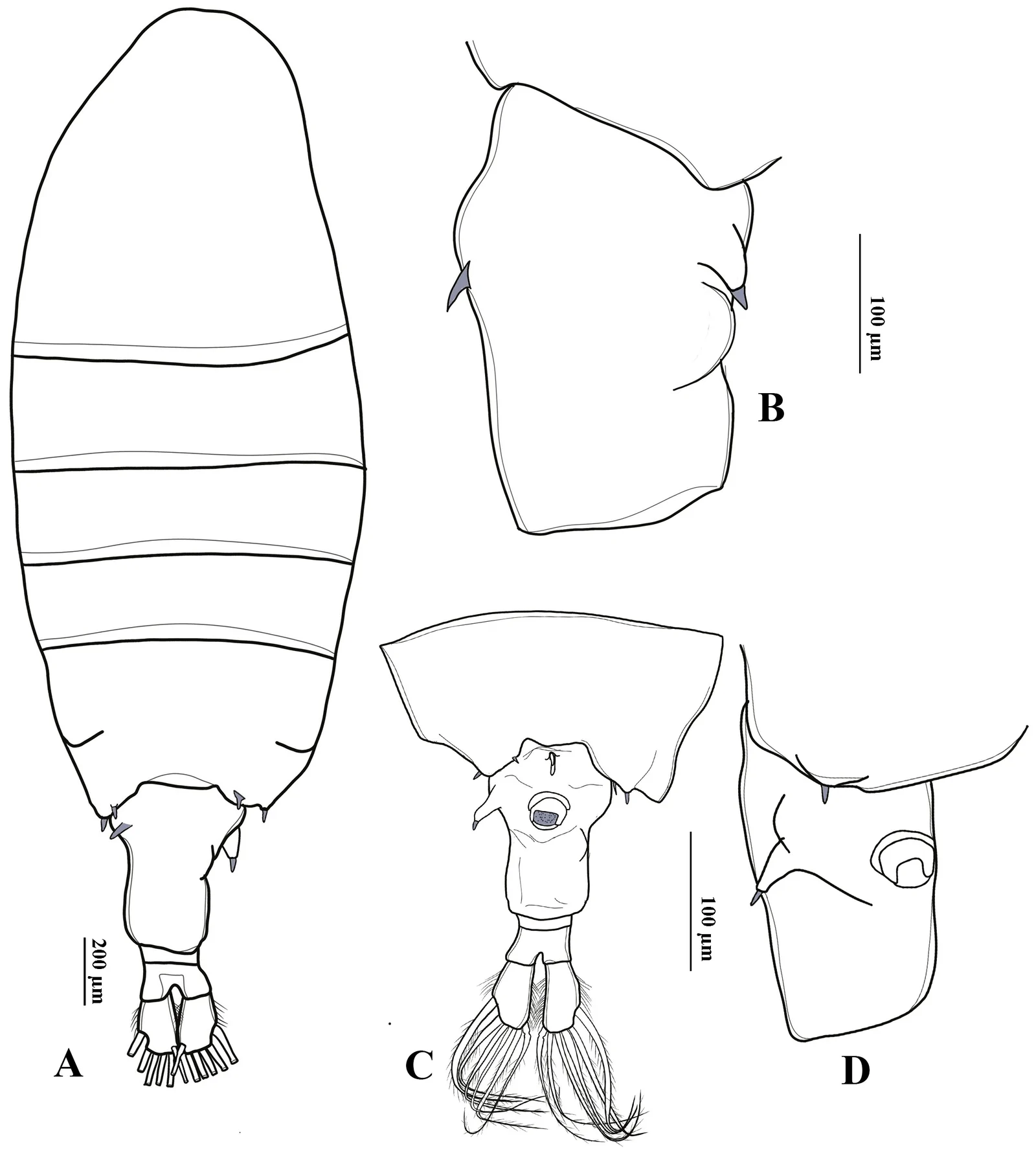
*Mongolodiaptomus rarus*, female. (A) Habitus, dorsal; (B) pediger 5 and genital double-somite, lateral (without caudal rami); (C) pediger 5, urosome and caudal rami, ventral; (D) pediger 5, lateral (without caudal rami).

Mandible (Fig. 6B): approximately four robustly chitinized teeth on dorsal side and one seta on dorsal coxal gnathobase. Basis featuring two serrated setae and two naked setae along the inner border. Enp bifurcated into two segments. Enp-1 possesses four setae on inner distal corner. Enp-2 possesses nine setae at apex and diminutive spinules around outer border. Exp four segmented. Exp-1–3, each having one seta on inner margin. Exp-4 with three naked, apical setae.

**Figure 10:**
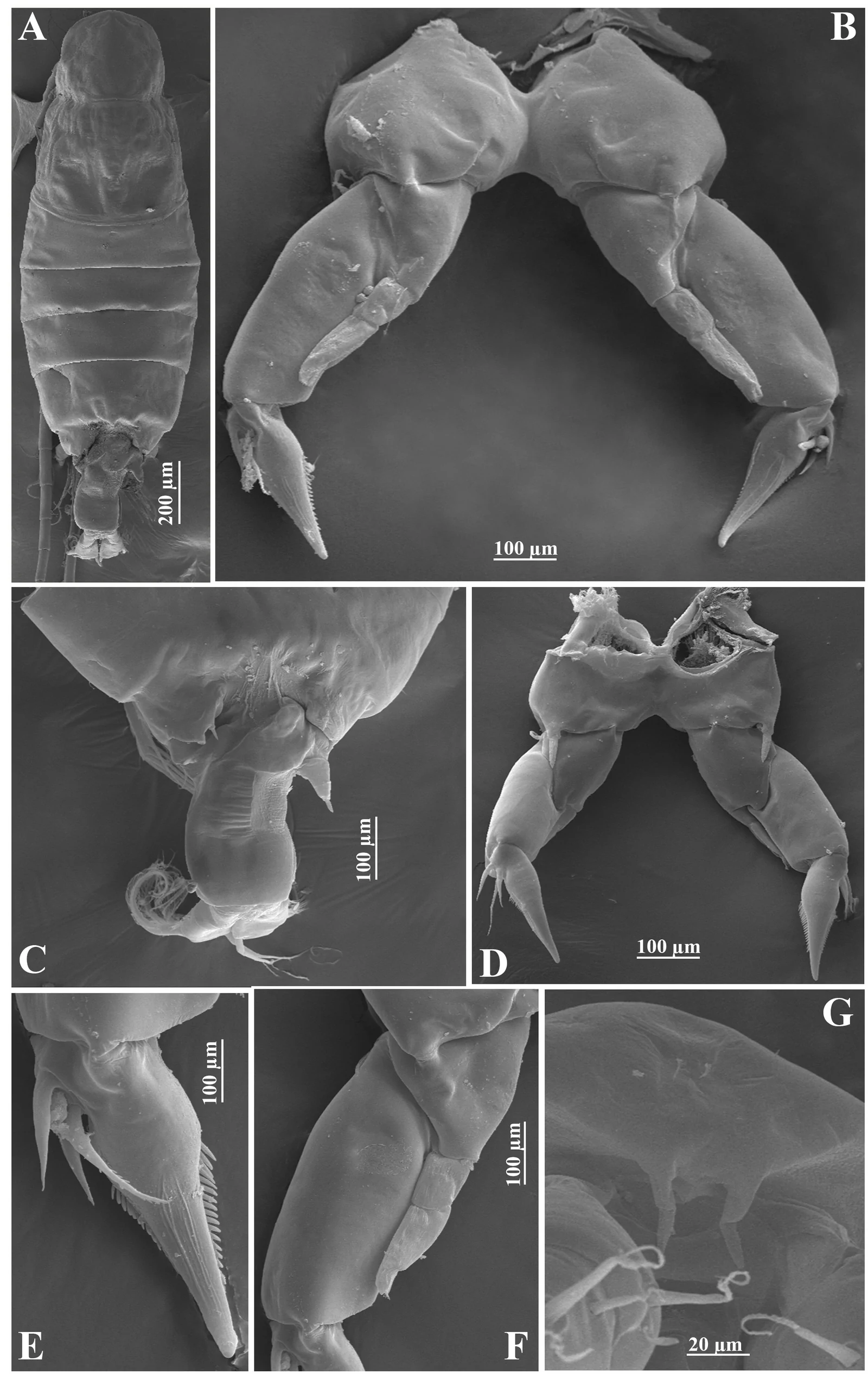
*Mongolodiaptomus rarus*, SEM photographs of female. (A) Habitus, dorsal; (B) P5, posterior; (C) pedigers 4–5, urosome, and caudal rami, dorsal; (D) P5, anterior; (E) distal part of left P5, posterior; (F) P5 Exp-1 –2, and Enp, posterior; (G) rostrum.

**Figure 11:**
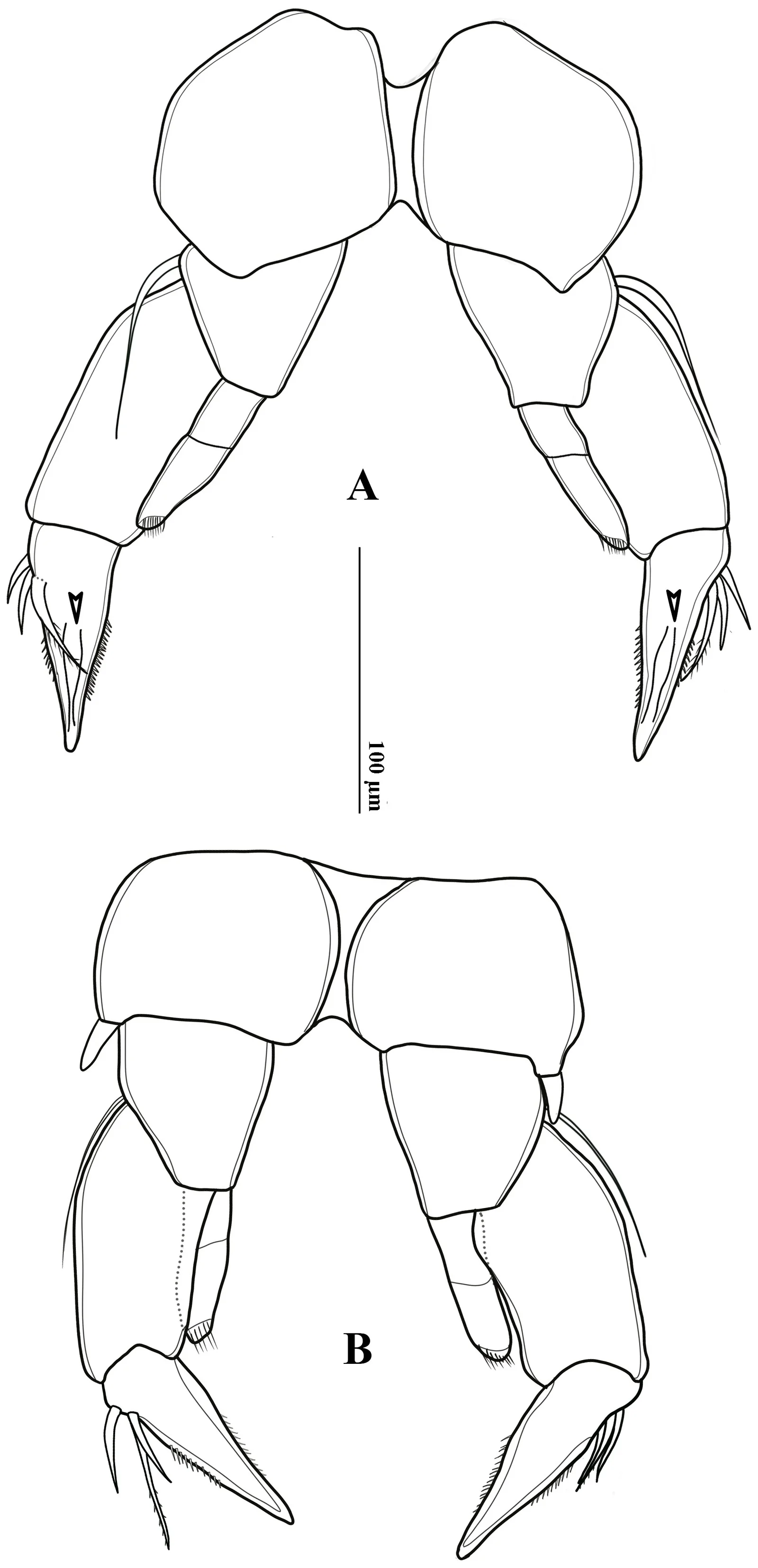
*Mongolodiaptomus rarus*, female P5. (A) Posterior (black arrows indicate longitudinal ridges); (B) anterior.

Maxillule (Fig. 6C): praecoxal arthrite with seven strong setae laterally and four slender submarginal setae. Coxal endite with three thin setae; coxal epipodite with nine setae. Basal endites fused to segment bearing them: proximal and distal endites, each with four setae apically; basal exopodite with one short seta. Enp-1 with four inner setae and eight apical setae, proximal segment fused to basis. Exp unsegmented with three outer setae, three apical setae, and hairy inner margin.

Maxilla (Fig. 6D): praecoxa fused to coxa. Proximal and distal endites on praecoxa, each with three setae apically. Two coxal endites, each with three setae apically. Allobasis with four setae apically. Enp two-segmented, each with three setae.

Maxilliped (Fig. 6E): four medial lobes on syncoxa with setal formulae 1, 2, 3, and 4, respectively; subdistal inner margin produced into a spherical lobe with a patch of tiny spinules. Basis with three setae along distal inner margin, with a row of tiny spinules proximally. Enp six-segmented, with 2, 3, 2, 2, 2, and 4 setae, respectively.

Swimming legs (P1–P4) (Figs. 7A–7D) biramous, with three-segmented rami, except for two-segmented Enp on P1. Exp longer than Enp. Each coxa on P1–P4 with one pinnate seta at innermost distal corner. P1–P3 bases without setae. P4 basis with one outer seta (Fig. 7D). P2 Enp-2 with Schmeil’s organ on posterior surface. Armature formulae of P1–P4 as in Table 4.

**P5** (Figs. 3C, 3F, 3H, 8A–8B) asymmetrical. Right P5: coxa somewhat rectangular, wider than long, slightly produced at distal inner corner into small, roughly triangular plate (Fig. 8A). Coxal spine long and slender, slightly curved, acuminate, and mounted on a small lobe (Fig. 3C, arrow). Basis roughly rectangular and sturdily built (Fig. 3F), with outer and inner margins convex and about 1.3 times as long as wide. Basis armed with a prominent ‘spurlike process’ near distal outer corner of posterior surface (Fig. 8A), a thin hyaline membrane along middle inner margin (Figs. 3G, 8A–8B), two small, asymmetrical chitinous projections located approximately at midpoint of proximal half of its segment, and a small seta located at two-thirds of outer margin. Exp two-segmented; Exp-1 small, shorter than wide, about 0.9 times as long as wide; two projection knobs on posterior surface; distal outer corner produced. Exp-2 prolonged oval shape, approximately 1.9 times as long as wide; inner margin convex and with a sub-globular knob located near proximal inner margin. Outer margin virtually straight, armed with one principal lateral spine situated at slightly proximal mid-outer margin, one square accessory knob inserted at proximal third of outer margin, and a tiny accessory spine located at distal outer margin, near the proximal part of end claw. Principal lateral spine short, about one-third length of its segment, closely attached to outer margin, and pointed to distal part of its segment (Figs. 3C, 3H). End claw slender with a strong proximal area, about 1.8 times as long as length of Exp-2, with internally serrated margin; proximal half smoothly curved, and distal half nearly straight and gradually tapers to an acuminate tip. Enp one-segmented, conical (Figs. 3C, 3F, 3H), and progressively tapers to the distal end, reaching to proximal third of Exp-2, apex with a row of spinules.

Left P5 (Figs. 3C, 3E–3G, 8A–8B): coxa somewhat squarish, armed with a long, slender seta inserted on small lobe near distal inner margin of posterior surface, extending beyond midpoint of basis. Basis robust, about 1.3 times as long as wide, with a small, thin hyaline lamella on mid-length of inner margin and a thin sensory seta near distal outer corner (Figs. 3F, 3G, 8A–8B). Exp three-segmented: Exp-1 conical, longer than wide, inner margin concave, with hairy lobe on inner margin. Exp-2 with a specialized crescent-shaped lobe of spinules at about proximal 2/3 length of segment (Fig. 3E). Exp-3 reduced, with a short, digitiform apical process, covered with a minute hyaline tip. Seta slender, spiniform, and slightly curved. Enp one-segmented; almost cylindrical, slightly shorter than Exp-1, with a row of spinulules on distal end.

**Table 2:** Armature formula of the left male antennule of *Mongolodiaptomus rarus*. The number of setae (Arabic numerals), aesthetascs (ae), and spines (sp) is given. The Roman numerals refer to segment numbers.

	Segment number												
	I	II	III	IV	V	VI	VII	VIII	IX	X	XI	XII	XIII
Number of elements	1+ae	3+ae	1+ae	1	1+ae	1	1+ae	1+sp	2+ae	1	1	1+ae+sp	1
	XIV	XV	XVI	XVII	XVIII	XIX	XX	XXI	XXII	XXIII	XXIV	XXV	
Number of elements	1+ae	1	1+ae	1	1	1+ae	1	1	2	2	2	4+ae	

**Table 3:** Armature formula of the right male antennule of *Mongolodiaptomus rarus*. The number of setae (Arabic numerals), aesthetascs (ae), spines (sp), and spiniform process (spr) is given. The Roman numerals refer to segment numbers.

	Segment number										
	I	II	III	IV	V	VI	VII	VIII	IX	X	XI
Number of elements	1+ae	3+ae	1+ae	1+ae	1+ae	1	1+ae	1+sp	2+ae	1+sp	1+sp
	XII	XIII	XIV	XV	XVI	XVII	XVIII	XIX	XX	XXI	XXII
Number of elements	1+ae+sp	1+ae+spr	2+ae+spr	2+ae+sp	2+ae+sp	2+spr	1+spr	2+ae+spr	3+spr	2	4+ae

**Table 4:** Armature formula for the swimming legs (P1–P4) of *Mongolodiaptomus rarus*. The quantity of setae (Arabic numerals) and spines (Roman numerals) is represented in the following sequence: outer-inner margin or outer-apical-inner margin.

	**Coxa**	**Basis**	**Exp**			**Enp**		
			**1**	**2**	**3**	**1**	**2**	**3**
P1	0-1	0-0	I-1	0-1	I-3-2	0-1	I-2-3	- - -
P2	0-1	0-0	I-1	I-1	I-3-3	0-1	0-2	2-2-3
P3	0-1	0-0	I-1	I-1	I-3-3	0-1	0-2	2-2-3
P4	0-1	1-0	I-1	I-1	I-3-3	0-1	0-2	2-2-3

**Description of adult female.** Total body length, measured from anterior margin of rostrum to posterior margin of caudal rami, 1.35–1.49 mm (mean = 1.40 mm, *n* = 5) (Figs. 9–11). Prosome: urosome ratio about 2.9: 1. Prosome similar to that of male. Rostrum symmetrical, acutely pointed (Fig. 10G). Fourth and fifth pedigerous somites incompletely fused. Pdg5 with asymmetrical posterolateral wings (Figs. 9A, 10A); right wing rounded and wider than left wing; left wing triangular and longer than right wing; each wing with one inner and one outer spine on posterolateral margins. Urosome three-segmented, with asymmetrical genital double-somite (Figs. 9A–9D). Genital double-somite longer than urosomite 2, anal somite, and caudal rami combined (Figs. 9A, 9C); one-third of area of left and right proximal regions dilated; left side with one dorsolateral spine on dilated region, and this spine greater in size than other genital spines (Fig. 9A); and right side with a posterolaterally directed conical process (Figs. 9A, 9C, 10C), reaching beyond about one-third of its segment. A pair of gonopores situated beneath a genital operculum on proximal, mid-ventral portion (Figs. 9C, 9D). Urosomite 2 symmetrical and shorter than wide. Anal somite symmetrical, shorter than length of caudal rami (Figs. 9A, 9C). Caudal rami parallel and symmetrical; each ramus enlarged on distal end, and both rami with hairy inner and outer margins (Figs. 9A, 9C). Each ramus with 6 setae (setae II–VII): setae II–VI plumose, anterolateral (II) seta with a smooth region on outer margin proximally, terminal setae (setae IV and V) without a fracture plane, and dorsal seta (VII) articulated, bare, and longest.

Antennule symmetrical; left antennule, antenna, mouthparts, and P1–P4 as in male.

P5 symmetrical (Figs. 10B, 10D–10F, 11A, 11B). Intercoxal sclerite narrow and symmetrical. Coxa with blunt spine on protuberance at distolateral corner in anterior surface (Fig. 11B). Basis with a thin, bare seta on distolateral margin, reaching beyond middle of Exp-1. Exp three-segmented; Exp-1 rectangular, about 2.0 times as long as wide, somewhat longer than Enp. Exp-2 triangular, with a row of strong spinules along both margins, with longitudinal grooves (conveyor canals) on posterior view (Figs. 10E, 11A), and a small outer spine proximally. Exp-3 reduced into a small knob on Exp-2, armed with two unequal spiniform setae apically (Figs. 10D, 11A, 11B). Enp two-segmented (Figs. 10F, 11A, 11B), subconical, about 3/4 times as long as Exp-1, with an obliquely truncate and finely spinulose apex.

## Discussion

### Interspecies relationships

*Mongolodiaptomus rarus* is assigned to this genus due to its compliance with the generic criteria established by Ranga Reddy, Sanoamuang & Dumont (2000): the second exopod segment of the male right P5 has three lateral spines; a principal spine inserts on the mid-outer margin, whereas two small spinous processes are each located proximally as well as distally. According to the classification of *Mongolodiaptomus* members based on the male characters by Sanoamuang & Koompoot (2024), the 17 known species globally have been updated into four species groups: (1) the *birulai* group, comprising *M. birulai* (Rylov, 1922), *M. botulifer* (Kiefer, 1974), *M. formosanus* Kiefer, 1937, *M. longiserratus* Chaicharoen & Sanoamuang, 2025, *M. malaindosinensis* (Lai & Fernando, 1978), and *M. parabirulai* Chaicharoen & Sanoamuang, 2025; (2) the *mephistopheles* group, comprising *M. mephistopheles* (Brehm, 1933), *M. nakhonphanomensis* Watiroyram, Monongdern & Koompoot, 2025, *M. pectinidactylus* (Shen & Tai, 1964), and *M. uenoi* (Kikuchi, 1936); (3) the *loeiensis* group, comprising *M. loeiensis* Watiroyram & Sanoamuang, 2017, *M. mekongensis* Sanoamuang & Watiroyram, 2018, and *M. phutakaensis* Sanoamuang & Koompoot, 2024; and (4) the *gladiolus* group, comprising *M. calcarus* (Shen & Tai, 1965), *M. dumonti* Sanoamuang, 2001, *M. gladiolus* (Shen & Lee, 1963), and *M. rarus* (Ranga Reddy, Sanoamuang & Dumont, 1998).

Within the *gladiolus* species group, four members exhibit the following male characteristics: (1) the spinous process on the antepenultimate segment of the right antennule is comb-like; (2) segment 16 of the right antennule possesses a spine; (3) the inner distal margin of the P5 intercoxal plate is either unproduced or barely formed; (4) the right P5 Exp-1 lacks an acute process on the outer distal margin; and (5) the right P5 Enp is conical or slender in shape, measuring approximately or less than half the length of the Exp-2 segment. However, the male morphological structures of *M. rarus* display the closest resemblance to those of *M. calcarus* (Table 5); both species possess the following characteristics: (1) asymmetrical caudal rami, with the right ramus longer than the left; (2) a ‘spurlike process’ on the posterior surface of the right P5 basis near the distal outer corner; (3) a concave or straight outer margin of the right P5 Exp-2, contrasting with the irregular outer margin found in the other two species of this group (*M. dumonti, M. gladiolus*); and (4) a narrow or thin hyaline lamella along the inner margin of the left P5 basis. The females of *M. rarus* and *M. calcarus* share the following features (Table 6): (1) the right distal corner of the genital double-somite is not expanded; (2) the seta on the P5 basis is longer than half the length of the Exp-1; and (3) the Enp is two-segmented.

**Table 5:** Comparison of male morphological characters of members belonging to the *Mongolodiaptomus gladiolus* species group. Members of this group are *M. calcarus, M. dumonti, M. gladiolus, and M. rarus.*

Male characters	***M. calcarus***	***M. dumonti***	***M. gladiolus***	***M. rarus***
Urosomites 2 and 3	With ventral hairs	With ventral hairs	No data	Without ventral hairs
Caudal rami	Asymmetrical, right ramus longer than left one	Asymmetrical, right ramus longer than left one	Symmetrical	Asymmetrical, right ramus slightly longer than left one
Ventral surface of right caudal ramus	With two dissimilar (small and large arising from lobed structure) chitinous teeth, both located at proximal inner corner	With two (small bilobed and slightly larger triangle) chitinous teeth, both located at proximal middle surface	Without chitinous structure	With one triangular-shaped chitinous tooth, located at distal middle surface
Inner margin of right P5 basis	Without hyaline membrane	Without hyaline membrane	Without hyaline membrane	With hyaline membrane
Posterior surface of right P5 basis	With a ‘spurlike process’ near distal outer corner	With a ‘spurlike process’ on mid-distal margin	Without ‘spurlike process’	With a ‘spurlike process’ near distal outer corner
Shape of right P5 Exp-2	Inner margin convex and nearly concave outer margin	Inner margin convex and irregular outer margin; enlarged at proximal 1/3 and narrowed at distal 2/3 length	Inner margin convex and irregular outer margin; enlarged at proximal 1/3 and narrowed at distal 2/3 length	Prolonged oval shape, inner margin convex, and nearly straight outer margin
Principal lateral spine of right P5 Exp-2	Robust, moderately long, and curved, pointing toward posterolateral direction, located at distal to mid-length of outer margin	Robust, long, and straight, pointing toward posterolateral direction, located at distal 3/4 length of outer margin	Slender, short, and curved, pointing toward posterolateral direction, located at distal to middle of outer margin	Robust, short, curved, and closely attached to outer margin, pointing toward distal direction, located at proximal mid-outer margin
Right P5 end claw	Unusually thick, short, and sickle-shaped	Slender, long, and sickle-shaped	Slender, long, and sickle-shaped	Slender, long, and sickle-shaped
Inner margin of left P5 basis	With narrow hyaline lamella and dilated distally in lateral view	With narrow hyaline lamella and dilated distally in lateral view	Without hyaline lamella	With thin hyaline lamella on mid-length of margin
Inner margin of left P5 Exp-2	Proximal half with a field of spinules, distal half serrate	Proximal half with a field of spinules	Proximal half with a field of spinules	Proximal 2/3 length of segment with a specialized crescent-shaped lobe of spinules

**Table 6:** Comparison of female morphological characters of members belonging to the *Mongolodiaptomus gladiolus* species group. Members of this group are *M. calcarus, M. dumonti, M. gladiolus, and M. rarus*.

**Female characters**	***M. calcarus***	***M. dumonti***	***M. gladiolus***	***M. rarus***
Lateral wings on Pdg 5 (left: right)	Almost symmetrical	Asymmetrical	Asymmetrical	Asymmetrical
Genital double-somite	Right proximal region without posterolateral process	Right proximal region without posterolateral process	Right proximal region with a moderately developed posterolateral process	Right proximal region with a moderately developed posterolateral process
Right distal corner of genital double-somite	Not expanded	Not expanded	Distinctly expanded	Not expanded
Seta on P5 basis	Longer than half length of Exp-1	Longer than half length of Exp-1	Shorter than half length of Exp-1	Longer than half length of Exp-1
P5 Exp-3	Distinct	Distinct	Inarticulate (fused with Exp-2)	Inarticulate (fused with Exp-2)
P5 Enp	Two-segmented	Two-segmented	One-segmented	Two-segmented

Nevertheless, *M. rarus* is considered to be distinct from *M. calcarus* in various other male features (Table 5): (1) the urosomites 2 and 3 lack ventral hairs in *M. rarus* but possess ventral hairs in *M. calcarus*; (2) the ventral surface of the right caudal ramus has one triangular-shaped chitinous tooth, located at the distal middle surface in *M. rarus*, but has two dissimilar chitinous teeth, both located at the proximal inner corner in *M. calcarus*; (3) the inner margin of the right P5 basis has a hyaline membrane in *M. rarus* but is without any hyaline membrane in *M. calcarus*; (4) the principal lateral spine of the right P5 Exp-2 is short, and closely attached to the outer margin, located at the proximal mid-outer margin in *M. rarus*, but is moderately long, located at the distal to mid-length of the outer margin in *M. calcarus*; (5) the sickle-shaped right P5 end claw is slender and long in *M. rarus* but unusually thick and short in *M. calcarus*; and (6) the proximal 2/3 length of the inner margin of the left P5 Exp-2 is armed with a specialized crescent-shaped lobe of spinules in *M. rarus,* but the proximal half has a field of spinules and the distal half has a serrated margin in *M. calcarus*.

Their female characters can be differentiated by (1) the lateral wings on Pdg 5 in *M. rarus*, which are asymmetrical but almost symmetrical in *M. calcarus*; (2) the right proximal region of the genital double-somite with a moderately developed posterolateral process in *M. rarus* but without a posterolateral process in *M. calcarus*; and (3) the P5 Exp-3, which is inarticulate (fused with Exp-2) in *M. rarus* but distinct in *M. calcarus* (Table 6).

### Morphological variability

Nevertheless, the single male holotype, which was designated by Ranga Reddy, Sanoamuang & Dumont (1998), has some following features that differ from our specimens. The spines and spiniform processes on the right male antennule in the original description are present on segments 8, 10, 11, 12, 13, 15, and 20 but absent on segments 14, 16, 17, 18, and 19, while all appendages on the previously mentioned segments are present in our redescription (Fig. 5). This can be explained by the fact that only the spine was defined previously, whereas the spiniform process was recently added, and it refers to a longer and thicker spinelike process. It is possible that the spiniform processes are not reported in the 1998 original description. However, the spine on segment 16 can be seen distinctively (Fig. 5B) on the redescription but not observed in the original description. The phenetic variability of the spiniform processes on the antepenultimate segment (segment 20) is demonstrated in this redescription by having four to five teeth (Figs. 5C1–5C3), but the number of teeth was not stated in the original description. Another morphological difference is the right P5 basis contains a thin hyaline membrane along the middle inner margin in the redescription but is absent in the original description.

### Ecology and Distribution

*Mongolodiaptomus rarus* occurs mostly in temporary-water habitats (Table 1), such as small ponds, pools, roadside canals, and rice paddies (Sanoamuang, 1999; Sanoamuang, 2001; Sanoamuang, 2004; Sanoamuang & Athibai, 2002; Watiroyram & Sanoamuang, 2017; Sanoamuang & Watiroyram, 2018; Sanoamuang & Watiroyram, 2019; Sanoamuang & Dabseepai, 2021). This taxon sometimes inhabits swamps, which are permanent-water habitats (Sanoamuang & Koompoot, 2024). It appears to be rare, restricted to a few localities of northeast Thailand, and has to date been recorded from 20 sites in five provinces (Khon Kaen, Nakhon Phanom, Nong Bua Lam Phu, Sakon Nakhon, and Udon Thani). This species appears to occur in the areas that are connected to the Phong and Songkhram Rivers (Fig. 1). Currently, *M. rarus* is endemic to northeast Thailand.

This species was found during May and September, over a temperature range of 25–38 °C, pH of 6.6–8.5, and conductivity of 2–299 µS cm^−1^. Information on the type of habitats, sampling dates, locations, water variables, and co-occurrence of diaptomid species is presented in Table 1. *M. rarus* co-occurs with 1–5 other species of diaptomid copepods in the same habitats. The most common co-occurring species with *M. rarus* are *Phyllodiaptomus praedictus* Dumont & Ranga Reddy, 1994, *Vietodiaptomus blachei* (Brehm, 1951), *M. calcarus* (Shen & Tai, 1965), *Neodiaptomus songkramensis* Sanoamuang & Athibai, 2002, and *M. botulifer* (Kiefer, 1974).

### Conservation of rare species

Most commonly found diaptomid species in Thailand, such as *Mongolodiaptomus botulifer, M. calcarus, M. dumonti, M. malaindosinensis, Vietodiaptomus blachei, Phyllodiaptomus praedictus, P. christineae* Dumont, Ranga Reddy & Sanoamuang, 1996, *Eodiaptomus sanoamuangae* Ranga Reddy & Dumont, 1998, *Neodiaptomus yangtsekiangensis* Mashiko, 1951, and *H. elegans* Kiefer, 1935, inhabit both ephemeral and perennial aquatic environments (Sanoamuang & Dabseepai, 2021); thus, they have more or less no risk for extinction. Nevertheless, certain uncommon or rare species, including *M. rarus, M. loeiensis, H. phuthaioram* Sanoamuang, 2004, *Neodiaptomus siamensis* Proogkiat & Sanoamuang, 2008, *N. songkhramensis* Sanoamuang & Athibai, 2002, *Phyllodiaptomus roietensis* Sanoamuang & Watiroyram, 2020, and *Paradiaptomus greeni* (Gurney, 1906) (Sanoamuang & Dabseepai, 2021); this study), primarily or exclusively inhabit temporary-water environments and are under unstable risk.

Temporary aquatic habitats are among the most endangered inland water ecosystems globally, facing threats from tourism, alterations in land use, road construction, eutrophication, and various anthropogenic factors that negatively impact the native aquatic fauna (Reid et al., 2002; Kulkarni et al., 2018). Our faunistic investigations done on the freshwater sampling sites of northeast Thailand during the past three decades (Dumont, Ranga Reddy & Sanoamuang, 1996; Ranga Reddy, Sanoamuang & Dumont, 1998; Ranga Reddy, Sanoamuang & Dumont, 2000; Sanoamuang, 1999; Sanoamuang, 2001; Sanoamuang, 2004; Sanoamuang & Yindee, 2001; Sanoamuang & Athibai, 2002; Sanoamuang & Sivongxay, 2004) have revealed that certain uncommon or rare species are currently difficult to find in the fields due to the disappearance of their previous temporary water habitats. The development of road lane expansions and agricultural crops are major factors that cause the disappearance of temporary-water habitats, particularly roadside ponds and small rice paddy pools. Previously, temporary ponds or canals were usually associated with the main roads or highways throughout the country. However, the recent development of modern highway construction has resulted in the disappearance of these temporary water bodies. In addition, many agricultural crops using modern mechanical tractors have been introduced so that small temporary ponds or tiny pools are replaced by larger permanent reservoirs.

Some tropical diaptomid copepods are known to produce diapause eggs, which are a specialized type of egg that is highly resistant to harsh environmental conditions, allowing them to survive unfavorable, ephemeral conditions in temporary habitats or during dry seasons in river floodplains. Thus, *M. rarus* and other uncommon or rare diaptomid species appear to have such a strategy for avoiding harmful conditions and synchronizing growth and reproduction to favorable biotic and abiotic conditions (Boonmak & Sanoamuang, 2024). However, to conserve these rare taxa from extinction, we need to communicate with local people and government officials about how to sustain such specialized and unique microhabitats in our future environment.

## Conclusions

We redescribe *Mongolodiaptomus rarus*, a rare, endemic diaptomid copepod, based on both male and female specimens from freshwater habitats in northeast Thailand. The morphology of *M. rarus* closely resembles *M. calcarus* but is nevertheless markedly dissimilar from its congeners, particularly due to the unique oval configuration of the male right P5 s exopod and the placement of the major lateral spine on this segment. Other distinctive characters are (1) the urosomites 2 and 3 lack ventral hairs; (2) the ventral surface of the right caudal ramus has one triangular-shaped chitinous tooth, located at the distal middle surface; (3) the inner margin of the right P5 basis has a hyaline membrane; and (4) the proximal 2/3 length of the inner margin of the left P5 Exp-2 is armed with a specialized crescent-shaped lobe of spinules. *M. rarus* predominantly occupies temporary-water environments, although it rarely exists in permanent-water habitats. Recent advancements in the construction of roads and the introduction of various agricultural crops have led to the loss of their habitats, necessitating the protection of this rare taxon. Increased sampling efforts in remote regions of Thailand and adjacent countries may provide additional reports of this rare taxon.

## Additional Information and Declarations

### Field Study Permissions

The following information was supplied relating to field study approvals (i.e., approving body and any reference numbers): Ethical review and approval were waived for this study because the copepod samples we collected from natural habitats were obtained before September 14, 2015, which is when the Animal for Scientific Purposes Act B.E. 2015 took effect under Thai law.

### Data Availability

The following information was supplied regarding data availability: This is a descriptive taxonomic work.

### New Species Registration

The following information was supplied regarding the registration of a newly described species: Publication LSID: urn:lsid:zoobank.org:pub:9BB5F803-0946-4893-94B5-9CF437C17AAA.
